# Temperate *Streptococcus thermophilus* phages expressing superinfection exclusion proteins of the Ltp type

**DOI:** 10.3389/fmicb.2014.00098

**Published:** 2014-03-13

**Authors:** Yahya Ali, Sabrina Koberg, Stefanie Heßner, Xingmin Sun, Björn Rabe, Angela Back, Horst Neve, Knut J. Heller

**Affiliations:** ^1^Department of Microbiology and Biotechnology, Max Rubner-Institut (Federal Research Institute of Nutrition and Food)Kiel, Germany; ^2^Medical Biology Department, Faculty of Medicine, Jazan UniversityJazan, Kingdom of Saudi Arabia; ^3^Department of Biotechnology, Agricultural Research Center, Animal Health Research InstituteCairo, Egypt

**Keywords:** *Streptococcus thermophilus*, prophage, superinfection exclusion, TP-J34, TP-778L, TP-EW, TP-DSM20617

## Abstract

Lipoprotein Ltp encoded by temperate *Streptococcus thermophilus* phage TP-J34 is the prototype of the wide-spread family of host cell surface-exposed lipoproteins involved in superinfection exclusion (sie). When screening for other *S. thermophilus* phages expressing this type of lipoprotein, three temperate phages—TP-EW, TP-DSM20617, and TP-778—were isolated. In this communication we present the total nucleotide sequences of TP-J34 and TP-778L. For TP-EW, a phage almost identical to TP-J34, besides the *ltp* gene only the two regions of deviation from TP-J34 DNA were analyzed: the gene encoding the tail protein causing an assembly defect in TP-J34 and the gene encoding the lysin, which in TP-EW contains an intron. For TP-DSM20617 only the sequence of the lysogeny module containing the *ltp* gene was determined. The region showed high homology to the same region of TP-778. For TP-778 we could show that absence of the *attR* region resulted in aberrant excision of phage DNA. The amino acid sequence of mature Ltp_TP-EW_ was shown to be identical to that of mature Ltp_TP-J34_, whereas the amino acid sequence of mature Ltp_TP-778_ was shown to differ from mature Ltp_TP-J34_ in eight amino acid positions. Ltp_TP-DSM20617_ was shown to differ from Ltp_TP-778_ in just one amino acid position. In contrast to Ltp_TP-J34_, Ltp_TP-778_ did not affect infection of lactococcal phage P008 instead increased activity against phage P001 was noticed.

## Introduction

Superinfection exclusion (sie) is generally known as a mechanism by which a prophage residing in a host cell prevents infection of the lysogenic host cell by other phage through blocking DNA injection (Donnelly-Wu et al., 1993). This protects the host from being lysed by the infecting and multiplying incoming phage, and hence the prophage will not be destroyed in the process of phage multiplication (McGrath et al., 2002; Mahony et al., 2008).

Sie has been mostly described for prophages of Gram-negative bacteria: P22 residing in *Salmonella typhimurium* (Hofer et al., 1995), Lambda-like phages in *Escherichia coli* (Cumby et al., 2012), and kappa-phage K139 in *Vibrio cholerae* (Nesper et al., 1999). Interestingly, sie has also been described for lytic T-even phages of *E. coli* (Lu and Henning, 1994). In Gram-positive bacteria, sie has been identified in prophages of corynebacteria (Groman and Rabin, 1982), *Lactococcus lactis* (McGrath et al., 2002), and *Streptococcus thermophilus* (Sun et al., 2006). One common feature of many of these proteins appears to be their targeting to the external side of the cytoplasmic membrane by either an N-terminal membrane-spanning helix (Mahony et al., 2008; Cumby et al., 2012) or a lipid-anchor (Sun et al., 2006). One exception appears to be the Glo protein of *Vibrio cholerae*, which has been described to a be soluble periplasmic protein (Nesper et al., 1999).

In temperate *S. thermophilus* phage TP-J34, a sie system is encoded by the *ltp* gene, residing within the lysogeny module. *ltp* is transcribed in the prophage state and encodes a lipoprotein, which is tethered to the outside of the cytoplasmic membrane, where it prevents injection of the DNA of the infecting phage into the cytoplasm of the host cell (Sun et al., 2006). Besides its rather weak activity against *S. thermophilus* phages, Ltp shows high activity against lactococcal phage P008 (Sun et al., 2006).

Ltp has been shown to consist of three different functional units: a lipid moiety for membrane anchoring, a serine-rich spacer region, and a repeat domain responsible for sie (Sun et al., 2006; Bebeacua et al., 2013). When expressed without its lipid-anchor, its host-range is extended to phages P335 and P001 belonging to different lactococcal phage species (Bebeacua et al., 2013). Thus, the active domain of Ltp may represent a broad-spectrum phage-resistance protein.

Genes encoding proteins with amino acid sequence similar to Ltp have been found to be scattered among Gram-positive bacteria and phages. No such gene has been described for *L. lactis* strains and phages, respectively (Sun et al., 2006), although lactococci and streptococci and their phages are very closely related (Proux et al., 2002). Within the 11 publicly available sequenced genomes of *S. thermophilus* phages 2972, 5093, 7201, 858, ALQ13.2, Abc2, DT1, O1205, Sfi11, Sfi19, Sfi21 <http://www.ncbi.nlm.nih.gov/genomes/GenomesGroup.cgi?opt=phage&taxid=10239&host=bacteria>, *ltp* determinants have not been identified. Phages O1205 (Stanley et al., 1997) and Sfi21 (Brüssow and Bruttin, 1995) are the only temperate among the 11 phages. However, they are closely related to the virulent *S. thermophilus* phages (Brüssow and Bruttin, 1995; Lucchini et al., 1999; Desiere et al., 2002). They all together may form just one species (Quiberoni et al., 2010). A differentiation of the 11 phages according to their DNA-packaging mechanism resulted in two sub-species (Quiberoni et al., 2010), represented by Sfi21 (*cos*-type) and Sfi11 (*pac*-type) (Proux et al., 2002). O1205 belongs to the *pac*-type (Stanley et al., 1997), indicating that the type of infection is of minor importance for the relatedness of phages.

To investigate the distribution and diversity of members of the Ltp protein family among strains of *S. thermophilus* and to analyze the relatedness of phages carrying an *ltp* gene, we screened among *S. thermophilus* strains for prophages carrying genes similar to *ltp*. For two temperate phages - TP-J34L and TP-778L, we analyzed the whole genome sequences. Of the two other phages, TP-EW and TP-DSM20617, we determined the sequences of some selected DNA regions: *ltp* gene for both phages, lysogeny module for TP-DSM20617, and putative host specificity gene and lysin gene for TP-EW. The two Ltp proteins of phages TP-J34 and TP778 were functionally compared and found to differentially inhibit lactococcal phages. The differences in inhibition are discussed with respect to the differences found in the amino acid sequences of the two Ltp proteins.

## Materials and methods

### Bacteria and phages

*S. thermophilus* strains used in this study were: J34 (lysogenic wild type), J34-6 (prophage-cured J34), SK778 (lysogenic wild type), DSM20617 (lysogenic wild type, German Collection of Microorganisms and Cell Cultures - DSMZ), and EW (lysogenic wild type).

The following phages were used: TP-J34 (wild type lysate, obtained by induction of the prophage) (Neve et al., 2003), TP-J34L (deletion derivative of TP-J34) (Neve et al., 2003), TP-778 (wild type lysate, obtained by induction of the prophage; this study), TP-778L (single plaque isolate from wild type lysate, this study), TP-DSM20617 (wild type lysate, obtained by induction of the prophage; this study), TP-EW (wild type lysate, obtained by induction of the prophage; this study).

The following lactococcal phages from our collection were used to test for infection-blocking activities of Ltp-derivatives: P197, P220, P624, P653, P684 (c2-species); P955, P957, P983, P993, P996 (936-species); P615 (P335-species). They had been assigned to species by electron microscopic inspection of their morphologies.

### Growth media, growth conditions, phage propagation, prophage induction, phage-curing, and relysogenization

*S. thermophilus* strains were routinely grown at 40°C in modified M17 medium containing lactose (th-LM17) (Krusch et al., 1987). For phage propagation, glycine-lysis medium was used: thM17 supplemented with 8 mM CaCl_2_ and 1% glycine (Sun et al., 2006). Prophage induction was carried out with UV-light or mitomycin C. For UV-light induction, cells from a growing culture in log-phase were harvested by centrifugation, re-suspended in ½ volume of 0.1 M MgSO4 and pumped through a quartz tube (internal diameter, 1.3 mm; length, 75 cm) placed under a laboratory 254 nm UV lamp (Schütt, Göttingen, Germany) at short distances (maximum 5 cm). Thereafter, the cell suspensions were mixed with another ½ volume of double-concentrated th-LM17 medium and incubated in the dark at 40°C. Induction was considered successful, when complete lysis was seen after ca. 3–4 h. For mitomycin C induction, different concentrations of mitomycin C (between 0.1 and 1 μg/ml) were added to growing cultures at early log-phase. Induction was considered successful, when turbidity increased for ca. 90 min after mitomycin C addition and then dropped to low turbidity levels.

Phage lysates were routinely centrifuged (Beckmann J2-21 centrifuge, 6000 rpm, 20 min, 4°C) and subsequently sterile filtered (nitrocellulose filters, pore size 0.45 μm).

Efficiency of plating was determined as described by Sun et al. (2006). Spot assays for determining the effects of Ltp-derivatives on phage infection were carried out by spotting 10 μl each of serial dilutions of phage lysates on agar plates overlaid with 0.75% top agar seeded with appropriate host bacteria.

All other relevant and specific information can be found in Neve et al. (2003).

### DNA techniques

Isolation of chromosomal DNA followed the method of Leenhouts et al. (1990) with some modifications. Ten ml th-LM17 medium (supplemented with 40 mM DL-threonine) was inoculated with *S. thermophilus*. Incubation proceeded at 40°C until an optical density at 620 nm (OD_620_) of ca. 0.8 was reached. From 2 ml of the culture, cells were sedimented by centrifugation (Eppendorf microcentrifuge) and washed once with 2 ml of bi-distilled water. The cells were resuspended in 0.5 ml buffer pH 8.0, containing 20% sucrose, 10 mM Tris-HCl, 10 mM EDTA, 50 mM NaCl, 2.5 mg lysozyme and 30 units mutanolysin. After incubation at 55°C for 10 min, 25 μl of 10% SDS and 60 μl of proteinase K were added. After mixing by inversion, incubation proceeded for 1 h at 60°C. Finally, DNA was taken up in 200 μl Tris-EDTA buffer of pH 8.0.

Phage DNA was isolated from CsCl-purified phage with subsequent phenol extraction following the procedure described by Sambrook and Russel (2000).

Restriction analyses were done according to Sambrook and Russel (2000). Enzymes and recommended buffers were purchased from New England Biolabs (Frankfurt, Germany).

Agarose gel electrophoresis and Southern blot analysis were carried out as described by Sambrook and Russel (2000).

For digoxigenin-labeling of DNA, the “DIG DNA Labeling Kit” of Roche Diagnostics (Mannheim, Germany) was applied, following the manual of the supplier.

PCR was carried out on an Eppendorf Mastercycler 5333 or on a Perkin Elmer GeneAmp PCR System 9600. Primers (Table 1) were purchased from MWG Biotech (Ebersberg, Germany). The following pipetting scheme was used: 5 μl 10 × (NH_4_)_2_SO_4_ buffer, 5 μl dNTPs (2 mM), 2 μl Tween 20 (2.5%), 1 μl of each of both primers (100 μM), DNA polymerase [10 parts Taq-polymerase (Quiagen, Hilden, Germany) plus 1 part Pfu-polymerase (Stratagene, Amsterdam, The Netherlands), diluted 1:5 with distilled water], 1 μl template-DNA, bi-distilled water 34 μl. PCR was carried out as “hot start” PCR (D'Aquila et al., 1991), starting with 5 min at 95°C for denaturation, holding at 80°C for addition of polymerase, followed by 30 cycles involving denaturation (95°C for 1 min), annealing (at mean Tm of primer pair for 1 min) and elongation (72°C for variable duration: ca. 1 min for 1 kb expected length). Finally, PCR concluded with an elongation at 72°C for 5 min.

**Table 1:** **PCR-primers used for amplification of genomic DNA**.

**Primer**	**Sequence [**5**′ → **3**′]**	**References**
D8	GGGTTGGAGCATTAGAAG	This study
D12	ACCAACTGAAATGCTACC	This study
D8+	GGGTTGGAGCATTAGAAGGTGGATC	This study
D12+	TCCTACCACCAACTGAAATGCTACC	This study
LYSup	GAACGAGCATTGAACTAC	This study
LYSdown	CAGTTCACGATACAGGTC	This study
terS-F	GCTCATTTGTGGGCTGTC	This study
terS-R	CAACGGTCTTACCTGCTC	This study
ltp-F	TAGCAACAGCGTAGTCAGC	This study
pri.C1-R	AAGCAAAGAGGTAGCAGAATC	This study
lys1	CACAAGCCTTAAAAGAGGCA	This study
3	CACAATCCTTCATCAAGC	Bruttin et al., 1997
4	GCAAGGTAAAGCTGCAC	Bruttin et al., 1997
Int.cro.2	TTTTTCTCCCATGCACTAACC	This study
MZ12.R	ATAGCAGATTATCGAATCGGTCAG	This study
8F	AGAGTTTGATCCTGGCTCAG	Beumer and Robinson, 2005
1525R	AAGGAGGTGATCCAGCC	Beumer and Robinson, 2005
B	GGCAAGCTTCGCTCTTGCTTGTTCTC	This study
D	GGCGAATTCTAGCAACAGCGTAGTCAGC	This study

An internal 384 bp fragment of *ltp* was amplified by PCR as follows. The reaction solution in the thermal cycler contained 10 μl of 10× PCR kit buffer (Appligene Oncor, USA), 10 μl of dNTP-mix (Appligene Oncor, USA), 4 μl of Tween-20, 1 μl of both primers B and D (100 pmol/ml), 5 μl (0.1 μg) of DNA, 66.5 μl of H_2_O and 2.5 μl of Taq DNA polymerase (1 unit/μl, Roche). Negative controls were set up similarly except that template DNA was omitted. Prior to cycling, the reaction mixture was heated to 95°C for 5 min, followed by 35 cycles of 30 s at 95°C, 30 s at 50°C, 30 s at 72°C and a final extension at 72°C for 7 min.

For “long-range” PCR (expected PCR products of up to ca. 4 kb), amplification was done following the “touchdown” protocol of Don et al. (1991). Primer pair D8+ and D12+ was applied. Annealing temperature in the first cycle was 10°C higher than the mean Tm of the primer pair. In the following 29 cycles, annealing temperature was reduced by 0.5°C per cycle. Finally, 10 cycles were added with an annealing temperature °C lower than the mean Tm of the primer pair. Elongation in that case was always 4 min.

Sequencing of the TP-J34 genome was done on a LI-COR 4200 system (MWG Biotech) according to the instructions of the supplier. Sequencing-PCR was done using the “Thermo Sequenase fluorescent labeled primer cycle sequencing kit with 7-deaza-dGTP (RPN 2438)” (Amersham Pharmacia Biotech, Freiburg, Germany), following the instructions of the supplier. Sequencing primers were labeled with fluorescence dye IRD800 (MWG Biotech). The sequence was completely determined for both DNA strands. It is available under EMBL accession number HE861935.1.

Sequencing of genomic DNA of TP-778L was done by AGOWA (Berlin, Germany) using 454 sequencing with an average coverage of approximately 20 fold. The sequence is available under EMBL accession number HG380752.1

For sequencing of terminal ends of the integrated prophage and host DNA regions flanking the insertion sites, the following primers were applied: primer pair primer4 (targeting the gene encoding 50S ribosomal protein L19) (Bruttin et al., 1997) and int.cro.2 (targeting the *cro* gene of temperate *Streptococcus* phages) for amplification of the left and primer pair lys.1 (targeting the lysin gene of temperate *Streptococcus* phages) and primer 3 (targeting an untranslated DNA region) (Bruttin et al., 1997) for amplification of the right flanking region. Both sequences are available under EMBL accession numbers HG917969 (left) and HG917970 (right).

The sequence of the DSM20617 prophage lysogeny module defined by primers 4 and Mz12.R binding sites was completely determined on both strands by primer walking. The sequence is available under EMBL accession number HG917971.

### Cloning of *ltp*_TP-778_

Using primers *ltp*-XbaI and *ltp*-HindIII binding upstream and downstream, respectively, the *ltp*_TP-778_ open reading frame was amplified by PCR. After restriction with the corresponding restriction enzymes the *ltp* orf was ligateded into XbaI/HindIII-cleaved pMG36e. After transformation into *L. lactis* Bu2-60, transformed cells were selected and plasmids extracted. By DNA sequencing plasmid pYAL1-3 was confirmed to be the correct construct.

### Sequence analysis

For identification of open reading frames “*orf* finder” <http://www.ncbi.nlm.nih.gov/gorf/gorf.html> and “Artemis” (Rutherford et al., 2000) were applied. To obtain an overview over the major directions of transcription, only *orfs* with coding capacities larger than 100 amino acids were considered in a first draft. Gaps between *orfs* were inspected for potential *orfs* as small as ca. 50 amino acids by searching for appropriate start codons in connection with potential ribosome binding sites. For annotation “blast” analyses were performed directly on the genes predicted by “orf finder” or “Artemis.”

tRNA genes were searched for by applying the “tRNAscan-SE” program of Lowe and Eddy (1997), and the “Tandem Repeat Finder” (Benson, 1999) was applied for searching for tandem repeats.

Functional assignment of gene products to protein families and identification of motifs of functional significance was done online <http://smart.embl-heidelberg.de/smart/set_mode.cgi?NORMAL=1> using SMART (Simple Modular Architecture research Tool) (Schultz et al., 1998; Letunic et al., 2009).

Dot plots were performed online <http://www.vivo.colostate.edu/molkit/dnadot/index.html>, (Maizel and Lenk, 1981) with the window size set to 13 and the mismatch limit set to 0.

For multiple sequence alignment, ClustalW at the EMBL-EBI website <http://www.ebi.ac.uk/Tools/msa/clustalw2/> (Larkin et al., 2007) or BLAST <http://blast.ncbi.nlm.nih.gov/Blast.cgi?PAGE_TYPE=BlastSearch&BLAST_SPEC=blast2seq&LINK_LOC=align2seq> (Altschul et al., 1990) was applied.

CRISPR spacer sequences were searched for at the “CRISPRs web server” by blasting phage genomic DNA sequences against the CRISPR database <http://crispr.u-psud.fr/crispr/BLAST/CRISPRsBlast.php> (Grissa et al., 2007).

## Results

**Figure 1:**
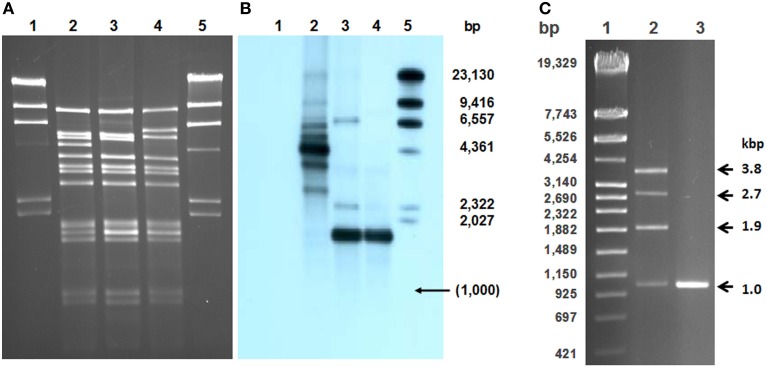
**Comparison of TP-J34, TP-J34L, and TP-EW genomic DNAs**. Agarose gel **(A)** and corresponding Southern blot **(B)** of HindIII-cleaved DNAs of TP-J34 (lane 2), TP-J34L (lane 3), and TP-EW (lane 4) hybridized with DIG-labeled 1 kb probe generated from 1.7 kb HindIII fragment of TP-J34L. Lanes 1 and 5: unlabeled and Dig-labeled λ-DNA, respectively. Sizes of restriction fragments of λ-DNA are shown in the right margin. Agarose gel **(C)** of PCR-products generated from TP-J34 (lane 2) and TP-J34L (lane 3) DNA with primer pair D8+ und D12+. Lane 1: DNA molecular weight marker IV (Roche Diagnostics GmbH, Mannheim, Germany), sizes are indicated in the left margin. Sizes of PCR products are shown in the right margin.

*S. thermophilus* temperate phage TP-J34 carrying an *ltp* gene has been described in some detail (Neve et al., 1998, 2003; Sun et al., 2006). Isolation of TP-778 has also been described (Neve et al., 2004). It has been identified as related to but considerably different from TP-J34 by subjecting DNAs extracted from 142 *S. thermophilus* strains and digested by HindIII to Southern blots using digoxigenin-labeled TP-J34 DNA as probe. In a further screening, more than 100 strains were tested by Southern hybridization with a probe generated from the *ltp*_TP-J34_ gene using primers B and D. Positive signals were obtained from three strains. Upon induction with mitomycin C two strains gave rise to phages with DNA restriction patterns identical to TP-J34 (data not shown). The third strain, *S. thermophilus* DSM20617, a strain from DSMZ collection which had been included in the screening, had originally been considered non-inducible (Sun, 2002). Only very recently it was shown to harbor an inducible prophage, named TP-DSM20617. TP-EW was identified as an inducible prophage in an *S. thermophilus* strain isolated from German yoghurt. Its DNA was found to give rise to restriction patterns highly similar to those of TP-J34, however, two restriction fragments in the HindIII restriction pattern differed from the TP-J34 pattern (see Figures 1A,B).

**Figure 2:**
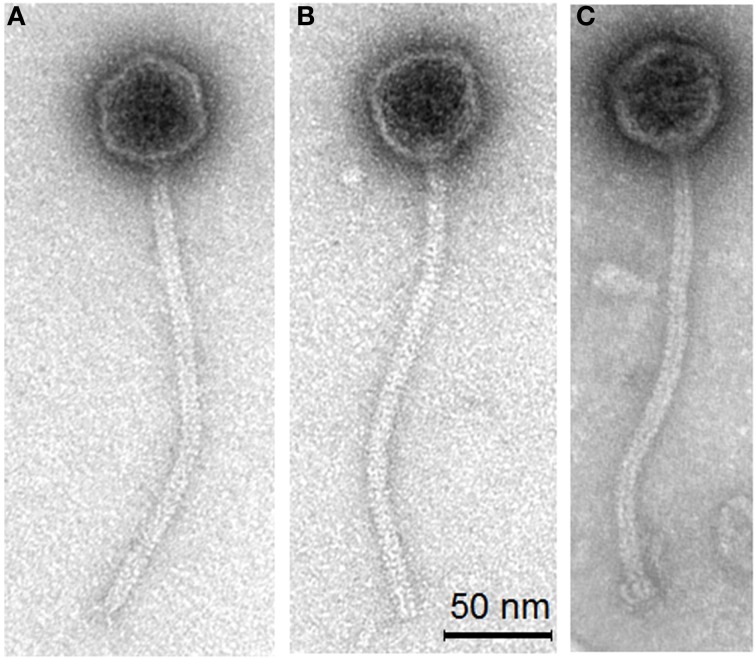
**Transmission electron micrographs of *S. thermophilus* phages TP-778L (A) propagated lytically on the prophage-cured derivative strain J34-2, phage TP-EW (B) and TP-DSM60217 (C) induced by mitomycin C from lysogenic *S. thermophilus* host strains EW and DSM20167, respectively**.

The morphologies of the three phages, TP-EW, TP-DSM20617, and TP-778L were almost identical to TP-J34 (Figure 2), the morphology of which—isometric head and long flexible tail of ca. 250 nm length—has been described already (Neve et al., 2003).

### Nucleotide sequences

We determined whole genome sequences for TP-J34 and TP-778L. In addition, left and right genome regions flanking prophage TP-778 were sequenced. For TP-EW, the two genome regions differing from those of TP-J34 (*orf48* and the lysin gene) were sequenced in addition to the *ltp* gene. For TP-DSM20617, only the genomic region corresponding to the lysogeny module of TP-J34, bearing the *ltp* sequence, was amplified from genomic DNA by PCR and sequenced.

**Figure 3:**
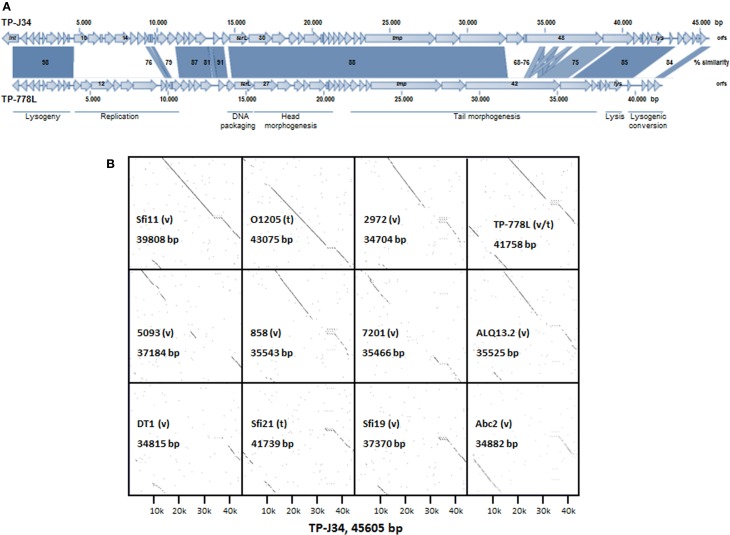
**(A)** Alignment of gene maps and functional gene regions of TP-J34 and TP-778L. On the genetic maps, genes, and direction of transcription are indicted by arrows (very small genes are shown as boxes, the directions of transcription correspond to adjacent genes). Numbers or gene abbreviations refer to *orf*s or genes as listed in Tables 3, 4. A scale indicating nucleotide positions is shown above the TP-J34 map. Approximate positions of functional regions (modules) are indicated by horizontal bars below the TP-778L map. Positions of CRISPR spacer sequences are indicated by dots above and below the maps of TP-J34 and TP-778L, respectively. **(B)** Dot plots of the TP-J34 nucleotide sequence compared to those of other *S. thermophilus* phages, including TP-778L. The horizontal line of each dot plot represents the 45,605 bp of TP-J34 DNA, whereas the vertical lines represent the numbers of bp for each phage, as indicated within each dot plot. Temperate (t) and virulent (v) phages are indicated.

In this section, we will address features TP-J34 and TP-778L genomes have in common, before we present in more detail those data, which are specific for the four phages and distinguish them from other *S. thermophilus* phages. TP-J34 and TP-778L DNAs share the same typical organization of functional modules characteristic for temperate *S. thermophilus* phages. Starting with the gene encoding the integrase, the order is: lysogeny module followed by modules for replication, DNA packaging, head morphogenesis, tail morphogenesis, lysis and finally lysogenic conversion (Figure 3A). While the lysogeny modules are transcribed from right to left, transcription of all other genes is from left to right. In none of the two genomes tRNA genes were detected. Sequences identical or highly similar to CRISPR spacer sequences in *S. thermophilus* strains were found in both genomes (Table 2). Their positions are indicated in Figure 3A. Orientations of the sequences are such that they correspond with the directions of transcription. Both phage genomes share with some other *S. thermophilus* phage genomes a site of a potential -1 translational frame-shift (Xu et al., 2004), which fuses *orf41* with *orf42* (TP-J34: bp 22942–23087) and *orf38* with *orf39* (TP-778L: bp 22560–22705), the two *orf*s in front of the gene encoding the tape measure protein (TMP). This frame-shift is known to result in formation of the tail assembly chaperone (Xu et al., 2013). TP-J34 has been shown to be a *pac*-type phage (Neve et al., 2003). By the same experimental approach, namely showing that minor DNA restriction bands were not affected by heat treatment of digested DNA, TP-778L was shown to be a *pac*-type phage as well. This corresponds with the rather high similarity seen between both large terminase units (Figure 3A).

**Table 2:** **CRISPR spacer sequences present in genomes of TP-J34 and TP-778L^a^**.

**Sequence ID/*S. thermophilus* strain phage**	**Spacer sequence^b^**	**Identity**	***E*-value**
**TP-J34**			
NC_008532_5_4 /LMD-9	agagtacaatattgtcctcattggagacac 5882 5911	1	7e-07
NC_008532_4_3 /LMD-9	catcataggcggaactggtaggatgtacac 44252 44281	1	7e-07
NC_006449_1_31 NC_006449_1_5 /CNRZ1066	gttggcaatgcaaacaacctttatgaaccg 40182 40211	1	7e-07
NC_017563_1_29 /NDO3	gaaagaatcggtcttctagatggattccaa 5245 5274	0.97	1e-04
NC_006449_1_6 /CNRZ1066	aaaggtggaacgttatcgcaaggaaataaa 33041 33070	0.97	1e-04
NC_006449_1_41 /CNRZ1066	atttgaaaaatgcacaacagcgtttgata 38388 38416	0.97	4e-04
**TP-778L**			
NC_017563_3_3 /ND03	cggacagcgataaatacactctatacagaga 12541 12571	1	2e-07
NC_017927_3_4 /MN-ZLW-002	attgacctattcaatgtatgggtcacgtaa 38358 38387	1	7e-07
NC_008532_2_3 /LMD-9	agtaatgatggtcggttatttttcagacat 36793 36822	0.97	1e-04
NC_006448_1_17 /LMG 18311	cattaaatcgcttgaagcagacattgaagc 4072 4101	0.97	1e-04
NC_008532_2_16 /LMD-9	aacagttactattaatcacgattcc 35406 35430	1	4e-04

a *Only sequences with E-values < 0.001 are shown*.

b *The phage sequences are shown with positions of first and last nucleotide*.

We compared the nucleotide sequence of TP-J34 with those of other *S. thermophilus* phages, for which complete genomes were available: O1205 (Stanley et al., 1997), Sfi21 and Sfi19 (Desiere et al., 1998), Sfi11 (Lucchini et al., 1999), 7201 (Stanley et al., 2000), DT1 (Tremblay and Moineau, 1999), 2972 (Levesque et al., 2005), 858 (Deveau et al., 2008), ALQ13.2, Abc2 (Guglielmotti et al., 2009), and 5093 (Mills et al., 2011). The alignments by DotPlot analysis are shown in Figure 3B. It appears that virulent phage Sfi11 and temperate phage TP-778 and O1205 are the most closely related to TP-J34. This is further reflected by the large number of putative gene products of these phages sharing highest homologies with those of TP-J34 (see Table 3).

**Table 3:** **Features of phage TP-J34 *orf*s and putative functions of their products**.

**ORF (gene)**	**DNA frame**	**Start**	**Stop**	**Size (aa)**	**SD sequence AAGGAGGT^a^**	**Predicted function/best match BLASTp result**	***E*-value**	**Match identity (%)**	**References, acc. no**.
1 (*int*)	−1	1080	1	359	TTGGGGGAttaaataa**ATG**	Integrase/ *S. thermophilus* phage Sfi21, Integrase/	0.0	100	Desiere et al., 1998
						359			
2 (*ltp*)	−3	1612	1184	142	ATGGAGGAaatttt**ATG**	Superinfection exclusion lipoprotein/ *Streptococcus parasanguinis*, prophage superinfection immunity protein	3e-42	51	-/
						152			WP_003010598
3	−1	2061	1693	122	AAAGTGAGaattt**ATG**	Putative metallo-proteinase/	1e-53	82	/
						*S. thermophilus* phage Sfi21, similar to cI-like repressor, metallo-proteinase motif			Desiere et al., 1998
4 (*crh*)	−1	2433	2068	121	AAGGAGAAagat**ATG**	Putative CI-repressor/	8e-21	55	/
						*S. thermophilus* phage Sfi21, CI-like repressor			Desiere et al., 1998
5 (*cro*)	+1	2602	2805	67	GAGGAGAAacaaa**ATG**	Putative Cro protein/	4e-28	91	/
						*S. thermophilus* phage 7201, Orf1, cro-like protein homolog			Stanley et al., 2000
6 (*ant*)	+2	2858	3574	238	AAGGATAAtac**ATG**	Putative antirepressor/	2e-129	98	/
						*S. thermophilus* phage Abc2, antirepressor protein			Guglielmotti et al., 2009
7	+1	3595	3876	93	ATAGGGGTtgaaaaagact**ATG**	-/	5e-47	98	/
						*S. thermophilus* phage Sfi21, Orf80			Desiere et al., 1998
8	+3	3936	4199	87	AAGGAATTaaa**ATG**	-/	3e-44	100	/
						*S. thermophilus* phage Sfi21, Orf87			Desiere et al., 1998
9	+2	4217	4357	46	GAGGAGAAacaaa**ATG**	-/	4.4	41	/
						S. pyogenes phage315.5, hypothetical protein SpyM3_1347			Beres et al., 2002
10	+1	4630	5517	295	GGGTGAGTctaaa**ATG**	-/	1e-142	99	/
						*S. thermophilus* phage 5093, putative primosome component			NC_012753
11	+2	5529	6311	260	AAAGGGGTtgact**ATG**	-/	5e-136	93	/
						*S. thermophilus* phage 5093, DnaC-like protein			NC_012753
12	+3	6308	6490	60	CAAGAGGAtgatgct**ATG**	-/	6e-27	100	/
						*S. thermophilus* phage 5093, hypothetical protein			NC_012753
13	+2	6615	7277	220	AAGGGAGAtaaa**ATG**	-/	1e-122	98	NC_012753
						*S. thermophilus* phage 5093, putative Erf protein			
14	+1	7279	8238	319	AAGGAGAActagc**ATG**	-/	7e-146	82	Guglielmotti et al., 2009
						*S. thermophilus* phage Abc2, hypothetical protein			
15	+1	8261	8710	149	CAGGAGAAaaaaac**ATG**	-/	1e-73	90	Guglielmotti et al., 2009
						*S. thermophilus* phage Abc2, single-stranded DNA binding protein			
16	+1	8719	9180	153	AAGGGAAAct**ATG**	-/	1e-82	97	Guglielmotti et al., 2009
						*S. thermophilus* phage Abc2, hypothetical protein			
17	+3	9177	9413	78	AAGGAGCTgga**ATG**	-/	3e-31	83	Stanley et al., 1997
						*S. thermophilus* temperate phage O1205, hypothetical protein			
18	+2	9404	9574	56	ATGGAGGAact**ATG**	-/	6e-19	85	Guglielmotti et al., 2009
						*S. thermophilus* phage Abc2, hypothetical protein			
19	+1	9571	9726	51	AAGGAGATtgattgaatt**ATG**	-/	2e-17	87	Desiere et al., 1998
						*S. thermophilus* phage Sfi21, hypothetical protein			
20	+3	9822	10028	68	AAAGAGGTaaattaa**ATG**	/	6e-11	62	ZP_01829218
						*Streptococcus pneumoniae*, hypothetical protein			
21	+3	10029	10670	213	AAAGAGGTggaatag**ATG**	/	8e-91	70	Beres et al., 2002
						*S. pyogenes* phage 2096.1, phage protein			
22	+1	10672	11217	181	TTGGAGAAaataaa**ATG**	/	5e-86	88	Lucchini et al., 1999
						*S. thermophilus* phage Sfi21, Orf178			
23	+3	11220	11732	170	AAAGAGGTgtaata**TTG**	/	4e-80	86	Deveau et al., 2008
						*S. thermophilus* phage 858, DNA binding protein (170aa)			
24	+1	11701	12018	105	AGGGAAGAtagtaa**ATG**	/	1e-43	94	Lucchini et al., 1999
						*S. thermophilus* phage Sfi18, gp99			
25	+2	12020	12463	147	GTAGAGGTaattaag**ATG**	/	1e-64	99	Lucchini et al., 1999
						*S. thermophilus* phage Sfi11, hypothetical protein			
26	+1	12469	13179	236	GCGTAGGAttc**ATG**	/	3e-117	86	Deveau et al., 2008
						*S. thermophilus* phage 858, Orf46			
27	+1	13615	14028	137	AGAGAGGTtagtaca**ATG**	/	5e-72	95	Lucchini et al., 1999
						*S. thermophilus* phage Sfi11, gp137, ArpU phage transcriptional regulator			
28 (*terS*)	+1	14177	14671	165	AAGGAGGTggatgt**ATT**	Putative terminase small subunit/	2e-111	98	Lucchini et al., 1999
						*S. thermophilus* phage Sfi11, gp172, putative terS product			
29 (*terL*)	+2	14658	15893	411	AAGGAGCTgtaaaca**ATG**	Putative terminase large subunit/	0	98	Lucchini et al., 1999
						*S. thermophilus* phage Sfi11, gp411, putative terL product			
30	+1	15899	17407	502	TAGGAGGaatg**ATG**	Putative portal protein/	0	99	Lucchini et al., 1999
						*S. thermophilus* phage Sfi11, gp502, portal protein			
31	+2	17404	18297	297	GAGAGGGTttatga**ATG**	/	5e-144	92	Lucchini et al., 1999
						*S. thermophilus* phage Sfi11, gp284, putative minor head protein; /			
32	+2	18486	19067	193	TAGGAGAAataa**ATG**	/	2e-105	99	Lucchini et al., 1999
						*S. thermophilus* phage Sfi11, gp193, putative scaffold protein			
33	+3	19087	19446	119	AAGGATTTtttaa**ATG**	/	6e-57	94	Stanley et al., 1997
						*S. thermophilus* temperate phage O1205, Orf30, putative structural protein			
34	+3	19465	20511	348	GAGGAGGAatattaaaac**ATG**	Putative major head protein/	0	97	Lucchini et al., 1999
						*S. thermophilus* phage Sfi11, gp348, major head protein			
35	+2	20523	20684	53	GAGGTGCTact**ATG**	/	3e-22	100	Lucchini et al., 1999
						*S. thermophilus* phage Sfi11, gp53			
36	+3	20696	21037	113	AGCGAGGTgtggc**ATG**	/	4e-57	96	Stanley et al., 1997
						*S. thermophilus* temperate phage O1205, hypothetisches Protein			
37	+2	21034	21348	104	GGTGAGGTgctatttct**ATG**	/	6e-54	100	Lucchini et al., 1999
						*S. thermophilus* phage Sfi11, gp104			
38	+2	21348	21692	114	AAGGTGGTtagata**ATG**	/	7e-60	100	Lucchini et al., 1999
						*S. thermophilus* phage Sfi11, gp114			
39	+1	21689	22075	128	TGGGATGAaac**ATG**	/	3e-71	100	Lucchini et al., 1999
						*S. thermophilus* phage Sfi11, gp128			
40	+3	22088	22594	168	TAGGAGGAaaaa**ATG**	Putative major tail protein/	7e-90	99	Stanley et al., 1997
						*S. thermophilus* temperate phage O1205, Orf37, major tail protein			
41	+2	22669	23022	117	TAGGAGTAaacaaaca**ATG**	/	2e-61	100	Lucchini et al., 1999
						*S. thermophilus* phage Sfi11, gp117			
42	+2	23085	23402	105	TACGAGGAattaatcacgaatgct**ATG**	/	1e-51	100	Lucchini et al., 1999
						*S. thermophilus* phage Sfi11, gp105			
43 (*tmp*)	+2	23392	27945	1517	AGAGAGGGgcttgctag**ATG**	Putative tape measure protein/	0	95	Lucchini et al., 1999
						*S. thermophilus* phage Sfi11, gp1510, putative minor tail protein			
44	+2	27945	29483	512	TGAGAGGTctcaatta**ATG**	/	0	94	Lucchini et al., 1999
						*S. thermophilus* phage Sfi11, gp512, putative minor tail protein			
45	+1	29483	32485	1000	AAGGTGGAttta**ATG**	/	0	97	Lucchini et al., 1999
						*S. thermophilus* phage Sfi11, gp1000, putative minor tail protein (Lysozyme and Chap domain)			
46	+1	32501	33622	373	TAGGAGGAattaaat**ATG**	/	0	98	Lucchini et al., 1999
						*S. thermophilus* phage Sfi11, gp373			
47	+3	33622	33795	57	TGTGAGGTgaatcaata**ATG**	/	7e-24	94	Lucchini et al., 1999
						*S. thermophilus* phage Sfi11, gp57			
48	+1	33773	38716	1647	GCGGAGTTaagta**ATG**	Putative host specificity protein /	0	72	Duplessis and Moineau, 2001
						*S. thermophilus* phage DT2, host specificity protein			
49	+2	38718	40727	669	TAGGAGAAgattaaa**ATG**	/	0	96	Lucchini et al., 1999
						*S. thermophilus* phage Sfi11, gp669, putative minor structural protein			
50	+1	40691	41092	133	AAAATGG**ATG**	/	1e-59	76	Lucchini et al., 1999
						*S. thermophilus* phage Sfi11, gp149			
51	+2	41112	41258	48	AAAGAGGAaaaagat**ATG**	/	9e-12	75	Desiere et al., 1998
						*S. thermophilus* phage Sfi21, hypothetical protein			
52	+2	41276	41599	107	AGGGATGTgtt**ATG**	/	3e-53	95	Lamothe et al., 2005
						*S. thermophilus* phage DT1, Orf23			
53 (*hol*)	+3	41605	41847	80	TGAGAGGAtaaagaca**ATG**	Putative holin/	4e-35	93	Stanley et al., 1997
						*S. thermophilus* temperate phage O1205, putative holin			
54 (*lys*)	+1	41849	42694	281	AGGAAGGAaaataat**ATG**	Putative lysin/	7e-141	90	Foley et al., 2000
						*S. thermophilus* phage S3B, putative lysin			
55	+2	42858	43256	132	AAGAAAAAcggctattgac**CTG**	/	5e-12	42	NZ_ABAA01000017
						*S. pneumoniae*, hypothetical protein (trans-membrane region)			
56	+1	43557	43892	111	AAAGAGGAaatgaa**ATG**	/	8e-55	100	Desiere et al., 1998
						*S. thermophilus* phage Sfi19, gp111			
57	+1	43914	44465	183	AAGGAGAAataaaaa**ATG**	/	5e-104	100	Lucchini et al., 1999
						*S. thermophilus* phage Sfi11, gp183			
58	+2	44491	44742	83	AACGAGGTgaaaaca**ATG**	/	6e-40	100	Lucchini et al., 1999
						*S. thermophilus* phage Sfi11, gp83			
59	+1	44768	44947	59	AAGCTTTAactgat**ATG**	/	1e-25	93	NC_012753
						*S. thermophilus* phage 5093, hypothetical protein			
60	+1	45006	45428	140	GAGGAAGTaaatgaa**ATG**	/	4e-63	85	NC_012753
						*S. thermophilus* phage 5093, hypothetical protein			

a *da Silva Oliveira et al., 2004*.

#### TP-J34 DNA

The nucleotide sequence was determined for DNA isolated from purified phage particles obtained by mitomycin C treatment of lysogenic *S. thermophilus* J34, as described before (Neve et al., 1998, 2003). TP-J34 DNA consists of 45,606 bp, and thus it is the largest of the *S. thermophilus* phage DNAs sequenced so far (http://www.ncbi.nlm.nih.gov/genomes/GenomesGroup.cgi?opt=virus&taxid=10699). It has a G+C content of 38.8%, which is similar to the 39% of its host (Bolotin et al., 2004). The sequence is accessible under NC_020197. Numbering of the TP-J34 sequence starts with the last nucleotide of the stop codon of the *int* gene.

Sixty *orf*s were predicted by the Artemis programme (Rutherford et al., 2000), all of which were considered as protein-encoding genes (Table 3) with protein sizes varying between 46 (*orf9*) and 1647 amino acids (*orf48*). The predominant start codon appears to be AUG (57 out of 60); one UUG (*orf23*), one AUU (*orf28*), and one CUG (*orf55*) were additionally predicted as start codons. AUU is a very unusual start codon (Blattner et al., 1997) normally coding for isoleucine. By repeated sequencing of PCR products generated with primers terS-F and terS-R using TP-J34 and TP-EW DNA, respectively, as templates, we excluded sequencing errors in this genomic region.

We have previously shown that upon induction of prophage TP-J34, mostly defective particles were released from the lysed host cells, and we have attributed the defect to a repeat region within *orf48* encoding the receptor binding protein (Neve et al., 2003). TP-J34L, an isolate forming clear plaques has been shown to have suffered a deletion of ca. 2.7 kb within the 4.4 kb HindIII fragment, thus reducing its size to 1.7 kb (Neve et al., 2003). In a Southern blot with HindIII-cleaved DNAs using a 1.0 kb PCR product (internal to the 1.7 kb HindIII fragment, obtained with primer pair D8/D12) of TP-J34L DNA as a probe, TP-J34 DNA extracted from lysates obtained by prophage induction yielded a major hybridization signal with the 4.4 kb fragment (Figures 1A,B). Two smaller signals at 3.5 and 2.6 kb were seen, indicating that the DNA was heterogeneous with respect to the 4.4 kb fragment, with 0.9 kb either one or two times deleted. As expected, TP-J34L DNA yielded a major signal at 1.7 kb. To confirm these results, the respective DNA regions of a TP-J34 lysate obtained by induction of the prophage and a TP-J34L lysate obtained by lytic propagation, were amplified by PCR, using primers D8+ and D12+ targeting sequences within the 4.4 kb HindIII fragment of TP-J34 but located outside of the repeat sequences. As expected, TP-J34L DNA gave rise to only one PCR product of ca. 1 kb. In case of the TP-J34 lysate, however, the DNA extracted yielded four products of ca. 1.0, 1.9, 2.8, and 3.7 kb (Figure 1C). This confirmed that TP-J34 DNA obtained by induction of the prophage was apparently heterogeneous with respect to the 4.4/1.7 kb HindIII fragment.

**Figure 4:**
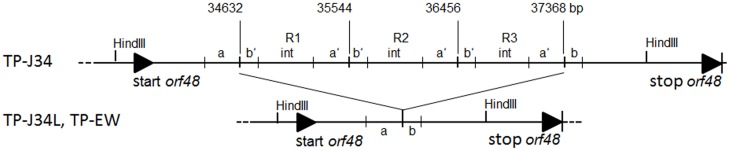
**Comparison of the genetic structure of the TP-J34 DNA region containing the triple repeat sequences R1–R3 with that of TP-J34L and TP-EW, respectively**. The bp numbers indicate the first bp of a repeat. “a” and “b” denote the regions with similarities to sequences within the repeats (marked as “a” and “b”). Sequences exclusively found within the three repeats are indicated as “int.” HindIII restriction sites flanking the 4.4 and 1.7 kb fragment of TP-J34 and TP-J34L/TP-EW, respectively are shown. Gene 48 start and stop are marked by solid triangles.

Inspection of the TP-J34 genome sequence in this region revealed a 912 bp repeat structure within *orf48* (Figure 4), located between genome positions 34,630 and 37,367. The triplicated sequence (3 × 912 bp) was found to be entirely in frame with the coding sequence of *orf48* encoding the putative host specificity protein. Theoretically, a gene product should be produced, which—according to the defective morphology of TP-J34—should be either inactive in the tail assembly process or physically unstable. We like to point out that when the TP-J34 prophage was induced and the resulting lysate was inspected by transmission electron microscopy after fractionation in a CsCl gradient, no tail structures were detected anywhere in the gradient (Neve et al., 2003).

**Figure 5:**
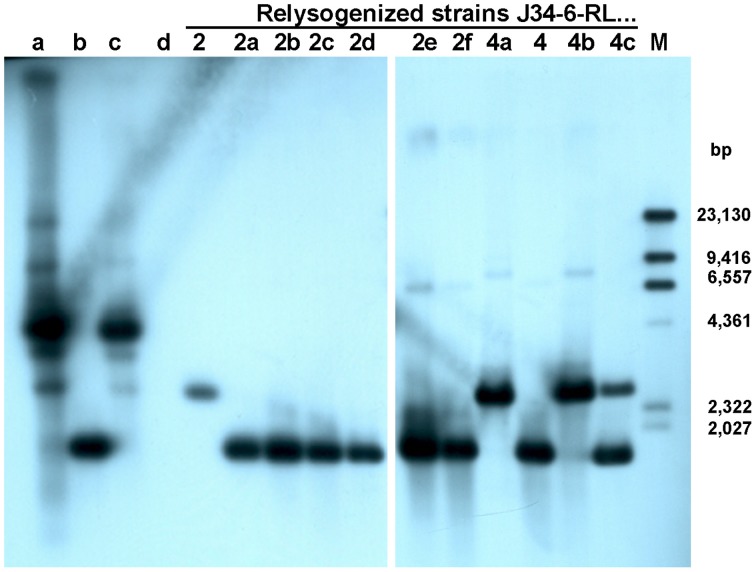
**Southern blot with DIG-labeled 1 kb probe of HindIII-cleaved phage and chromosomal DNA of eleven *S. thermophilus* strains relysogenized with TP-J34**. Lane a: TP-J34; lane b: TP-J34L; lane c: J34; lane d: J34-6 (no prophage, negative control); lane M: DIG-labeled, HindIII-cleaved λ DNA. Other lanes (from left to right): J34-RL2; J34-6-RL2a; J34-6-RL2b; J34-6-RL2c; J34-6-RL2d; J34-6-RL2e; J34-6-RL2f; J34-6-RL4a; J34-6-RL4; J34-6-RL4b; J34-6-RL4c. The sizes of the λ DNA bands are indicated in the right margin.

To genetically prove that the defect in *orf48* was responsible for the tail assembly defect, we used the lysate obtained by induction of the TP-J34 prophage, which contained mostly defective particles, for re-lysogenization of prophage-cured *S. thermophilus* J34-6. From 11 lysogens obtained, chromosomal DNA was isolated, restricted with HindIII and subjected to Southern blotting using the 1.0 kb PCR product of TP-J34L DNA as probe. Of the 11 strains, seven showed a hybridization signal at 1.7 kb, three a signal at 2.6 kb and one a strong signal at 1.7 and a weaker signal at 2.6 kb. Genomic DNA isolated from lysogenic *S. thermophilus* J34 yielded three signals at 2.6, 3.5, and 4.4 kb (Figure 5). Of two of the re-lysogenized strains, J34-6-RL2 (signal at 2.6 kb) and J34-6-RL4 (signal at 1.7 kb), prophage were induced with mitomycin C. The lysates obtained were subjected to electron microscopy and compared with lysates obtained by prophage induction of *S. thermophilus* J34 and by lytic propagation of TP-J34L. The vast majority of phage particles of TP-J34 and TP-J34-6-RL2 were defective, whereas about half of the TP-J34L and TP-J34-6-RL4 looked morphologically intact, when analyzed in the electron microscope. When measuring plaque formation, phage lysates of TP-J34L and TP-J34-6-RL4 each yielded ca. 10^8^ pfu/ml, while TP-J34 and TP-J34-6-RL-2 each yielded ca. 10^5^ pfu/ml. It thus appears that even an insertion of one 912 bp repeat is sufficient for inactivation of the tail assembly function of *orf48* gene product.

#### TP-778

The nucleotide sequence was determined for DNA isolated from CsCl-purified TP-778L, lytically propagated on *S. thermophilus* B106, as described in Materials and Methods. TP-778L DNA consists of 41,757 bp. It has a G+C content of 39%, which is identical to the 39% of its host (Bolotin et al., 2004). The sequence is accessible under NC_022776. Numbering of the TP-J34 sequence starts with the last nucleotide of the stop codon of the *int* gene. Of the 52 *orf*s predicted by the Artemis programme (Rutherford et al., 2000), all were considered as protein-encoding genes (Table 4) with protein sizes varying between 46 (*orf9*) and 2020 amino acids (*orf42*). The predominant start codon appears to be AUG (49 out of 52). Of the residual three, two appear to be GUG (*orf*s *16* and *19*) and one UUG (*orf43*).

**Table 4:** **Features of phage TP-778L *orf*s and putative functions of their products**.

**Orf (gene)**	**DNA-frame**	**Start**	**Stop**	**Size (aa)**	**SD sequence AAGGAGGT^a^**	**Predicted function/best match BLASTp result**	***E*-value**	**Match identity (%)**	**References, acc. no**.
1 (int)	−3	420	1	139	TTGGGGGAttaaataa**ATG**	Putative integrase/	2e-90	99	Desiere et al., 1998, NP_049990
						*Streptococcus thermophilus* phage *Sfi*21, integrase /			
						359			
2 (ltp)	−1	952	524	142	TGGTAGGAaatttt**ATG**	Putative superinfection exclusion lipoprotein/	3e-79	93	Neve et al., 1998, AAC03455
						*Streptococcus thermophilus* phage TP-J34/			
						142			
3	−2	1400	1032	122	AAGGAAAAgtgagaattt**ATG**	Putative metallo- proteinase motif/ *Streptococcus thermophilus* phage *Sfi*21, cI- like repressor/	4e-81	96	Desiere et al., 1998, NP_049992
						122			
4 (crh)	−2	1772	1407	121	AAGGAGAAagat**ATG**	Putative CI- repressor/	1e-80	100	Neve et al., 1998, AAC03457
						*Streptococcus thermophilus* phage TP-J34, putative cI-repressor homolog/			
						121			
5 (cro)	+3	1941	2144	67	GAGGAGAAacaaa**ATG**	Putative Cro protein/	3e-41	99	Neve et al., 1998, AAC03458
						*Streptococcus thermophilus* phage TP-J34, Cro-like regulatory protein/			
						67			
6 (ant)	+1	2197	2913	238	AGAAAGGAtaatac**ATG**	Putative antirepressor/	2e-175	99	Neve et al., 1998, AAC03459
						*Streptococcus thermophilus* phage TP-J34, P1-antirepressor homolog /			
						238			
7	+3	2934	3215	93	ATAGGGGTtgaaaaagact**ATG**	-/	1e-61	100	Neve et al., 1998, AAC03460
						*Streptococcus thermophilus* phage TP-J34, hypothetical protein/ 93			
8	+2	3275	3538	87	AAGGAATTaaa**ATG**	-/	6e-57	100	Desiere et al., 1998, NP_597801
						*Streptococcus thermophilus* phage *Sfi*21 Orf87, hypothetical protein *Sfi*21p33/			
						87			
9	+1	3556	3693	46	AAAGAGGAgaaacaaa**ATG**	-/	4e-26	100	This study
						*Streptococcus thermophilus* phage TP-J34, hypothetical protein/ 46			
10	+2	3932	4405	157	AAGGAGTAtaccataaaat**ATG**	*-/*	4e-88	84	Guglielmotti et al., 2009, YP_003344879
						*Streptococcus thermophilus* phage ALQ13.2, hypothetical protein/ 157			
11	+1	4402	5103	233	AAGGAGAAaccttaacataag**ATG**	-/	3e-168	99	Levesque et al., 2005, YP_238517
						*Streptococcus thermophiles* phage, putative replication initiation protein/			
						233			
12	+2	5060	6472	470	AAAGGGGTgtaaggtag**ATG**	-/	0.0	99	Deveau et al., 2008, YP_001686831
						*Streptococcus thermophilus* phage 858 Orf 37, putative helicase/			
						470			
13	+2	6479	6952	157	TTGGAGATaaaaaaac**ATG**	-/	3e-108	97	Deveau et al., 2008, YP_001686832
						*Streptococcus thermophilus* phage 858 Orf 38/			
						157			
14	+3	6957	7772	271	TTTGCCATtctaagact**ATG**	-/	0.0	99	Deveau et al., 2008, YP_001686833
						*Streptococcus thermophilus* phage 858 Orf 39, primase-polymerase domain/ 271			
15	+1	7741	9297	518	AAGGAGTTagatactaaac**ATG**	Putative primase/	0.0	92	Geng et al., 2011, YP_006561246
						*Streptococcus* phage YMC-2011, putative primase /			
						519			
16	+1	9541	9861	106	AGAAAGGTaaattttaa**GTG**	-/	1e-65	92	Deveau et al., 2008, YP_001686835
						*Streptococcus thermophilus* phage 858 Orf 41, VRR_NUC domain/			
						106			
17	+2	9845	10081	78	AAGGAAGCtttggatatagtaa**ATG**	-/	7e-42	87	Guglielmotti et al., 2009, YP_003347446
						*Streptococcus thermophilus* phage Abc2, hypothetical protein/			
						78			
18	+3	10098	10253	51	AAGATGGTagagtt**ATG**	-/	1e-23	84	Desiere et al., 1998, NP_049960
						*Streptococcus thermophilus* phage Sfi19 Orf 51; hypothetical protein Sfi19p40/			
						51			
19	+3	10254	10835	193	GAGGTGGAataa**GTG**	-/	9e-58	69	Guglielmotti et al., 2009, YP_003347451
						*Streptococcus thermophilus* phage Abc2, hypothetical protein/			
						166			
20	+3	10836	11348	170	GAAGAGGTtgaataa**ATG**	Putative DNA-binding protein/	6e-111	91	Mills et al., 2011, YP_002925093
						*Streptococcus thermophilus* phage 5093, DNA binding protein, HTH_XRE/			
						170			
21	+1	11317	11634	105	AGGGAAGAtagtaa**ATG**	-/	1e-65	95	This study
						*Streptococcus thermophilus* phage TP-J34, hypothetical protein/			
						105			
22	+2	11636	12079	147	GTAGAGGTaattaag**ATG**	-/	2e-103	99	This study
						*Streptococcus thermophilus* phage TP-J34, hypothetical /			
						147			
23	+1	12085	12795	236	GTGGGGGCgtaggattc**ATG**	/	7e-161	94	Stanley et al., 2000, NP_038319
						*Streptococcus thermophilus* phage 7201 Orf 18/			
						235			
24	+2	13232	13645	137	AGAGAGGGcagaaaa**ATG**	Putative transcriptional regulator/	2e-94	99	This study
						*Streptococcus thermophilus* phage TP-J34 Orf27, transcriptional regulator ArpU family/			
						137			
25 (terS)	+2	13766	14278	170	TTTGAGTTgtctttttttgattatgaa**ATG**	Putative terminase small subunit/	7e-85	86	Levesque et al., 2005, YP_001686797
						*Streptococcus thermophilus* phage 2972, terminase small subunit/			
						150			
26 (terL)	+3	14265	15500	411	AAGGAGCTgttagcg**ATG**	Putative terminase large subunit/	0.0	97	This study
						*Streptococcus thermophilus* phage TP-J34 Orf29, putative terminase large subunit /			
						411			
27	+2	15506	17014	502	TAGGAGGAatg**ATG**	Putative portal protein/	0.0	97	Deveau et al., 2008, YP_001686800
						*Streptococcus thermophilus* phage 858 orf6, putative portal protein/			
						502			
28	+1	17011	17904	297	GAGAGGGTtatga**ATG**	Putative head protein/	0.0	96	Levesque et al., 2005, YP_238489
						*Streptococcus thermophilus* phage 2972, head protein/			
						297			
29	+3	18096	18677	193	TAGGAGAAcaaa**ATG**	Putative scaffold protein/	7e-130	96	Levesque et al., 2005, YP_238490
						*Streptococcus thermophilus* phage 2972, scaffold protein/			
						193			
30	+1	18697	19056	119	AAGGAAATtttaa**ATG**	Putative head protein/	6e-75	97	Levesque et al., 2005, YP_238491
						*Streptococcus thermophilus* phage 2972, head protein/			
						119			
31	+1	19075	20121	348	GAGGAGGAacattaaaac**ATG**	Putative capsid protein/	0.0	98	Guglielmotti et al., 2009, YP_003344853
						*Streptococcus thermophilus* phage ALQ13, capsid/			
						348			
32	+3	20133	20294	53	TAAGAGGTactgat**ATG**	-/	5e-19	98	Guglielmotti et al., 2009, YP_003344854
						*Streptococcus thermophilus* phage ALQ13, hypothetical protein/			
						53			
33	+2	20309	20647	112	AGTGAGGTatggcgtg**ATG**	-/	3e-70	94	Stanley et al., 1997, NP_695111
						*Streptococcus thermophilus* phage 01205 Orf 33, hypothetical protein/			
						122			
34	+1	20644	20958	104	GGTgaggtgctatttct**ATG**	-/	7e-62	94	Levesque et al., 2005, YP_238495
						*Streptococcus thermophilus* phage 2972, hypothetical protein /			
						104			
35	+2	20960	21304	114	AAGGTGaTgaaataac**ATG**	-/	4e-72	94	Lucchini et al., 1999, NP_056684
						*Streptococcus thermophilus* phage Sfi11 Orf 114, hypothetical protein/			
						114			
36	+1	21289	21687	132	GAAGAGATggcgaa**ATG**	-/	1e-84	95	Guglielmotti et al., 2009, YP_003344858
						*Streptococcus thermophilus* phage ALQ13, hypothetical protein/			
						128			
37	+2	21701	22210	169	AATTAGGAGGAaaaa**ATG**	Putative tail protein/	9e-115	98	Levesque et al., 2005, YP_238498
						*Streptococcus thermophilus* phage 2972, tail protein/			
						169			
38	+3	22287	22640	117	TAGGAGTAaacaaaca**ATG**	-/	6e-78	99	Levesque et al., 2005, YP_238499
						*Streptococcus thermophilus* phage 2972, hypothetical protein /			
						117			
39	+2	22703	23020	105	GAGGAGTTaatcactaatgcc**ATG**	-/	2e-65	99	Levesque et al., 2005, YP_238500
						*Streptococcus thermophilus* phage 2972, hypothetical protein /			
						105			
40 (tmp)	+3	23010	27563	1517	AGAGAGGGgcttgctag**ATG**	Putative tape measure protein/	0.0	90	Stanley et al., 2000 NP_695118
						*Streptococcus thermophilus* phage O1205, putative tail protein/			
						1517			
41	+2	27563	29101	512	TGCGAGGTctaaatta**ATG**	Putative tail protein/	0.0	89	Lucchini et al., 1999, NP_056690
						*Streptococcus thermophilus* phage Sfi11, putative minor structural protein/			
						511			
42	+1	29101	35163	2020	AAGGTGGAttta**ATG**	Putative host specificity protein/	0.0	88	Deveau et al., 2008, YP_001686815
						*Streptococcus thermophilus* phage 858 Orf21,prophage tail protein /			
						1006			
43	+1	35164	37185	673	TAGGAGGTttttaa**TTG**	Putative tail protein/	0.0	89	Deveau et al., 2008, YP_001686816
						*Streptococcus thermophilus* phage 858 Orf22/			
						673			
44	+1	37201	37548	115	AAGAAGGAaaattc**ATG**	-/	6e-73	96	This study
						*Streptococcus thermophilus* phage TP-J34, hypothetical protein/			
						133			
45	+2	37568	37714	48	AAAGAGGAaaaagat**ATG**	-/	2e-24	100	This study
						*Streptococcus thermophilus* phage TP-J34, hypothetical protein/			
						48			
46	+1	37732	38055	107	TAGGAGGGatgtgtt**ATG**	-/	2e-71	100	This study
						*Streptococcus thermophilus* phage TP-J34, hypothetical protein/			
						107			
47 (hol)	+3	38064	38306	80	TGAGAGGAtaaataacaat**ATG**	Putative holin/	7e-44	93	Guglielmotti et al., 2009, YP_003347430
						*Streptococcus thermophilus* phage Abc2, holin/ 80			
48 (lys)	+1	38308	39153	281	AAGGAAGGaaaatagt**ATG**	Putative lysin/	8e-181	90	This study
						*Streptococcus thermophilus* phage TP-J34, putative lysin/			
						281			
49	+1	39499	39720	73	AAGATTGAaaacaaactagacgac**ATG**	-/	5e-46	100	Neve et al., 1998, AAC03448
						*Streptococcus thermophilus* phage TP-J34/			
						73			
50	+3	40419	40715	98	AGAGAGGTaaaaagaa**ATG**	-/	2e-38	66	WP_009730541
						*Streptococcus* sp. F0441, hypothetical protein/			
						101			
51	+2	40778	41200	140	AAGGAAGTat**ATG**	-/	5e-85	90	Desiere et al., 1998, NP_049988
						*Streptococcus thermophilus* phage Sfi21, hypothetical protein /			
						140			
52	+3	41202	41624	140	AAAGATGTaatctaaa**ATG**	-/	1e-81	87	Desiere et al., 1998, NP_049989
						*Streptococcus thermophilus* phage Sfi21, hypothetical protein /			
						140			

*^a^da Silva Oliveira et al., 2004*.

**Figure 6:**
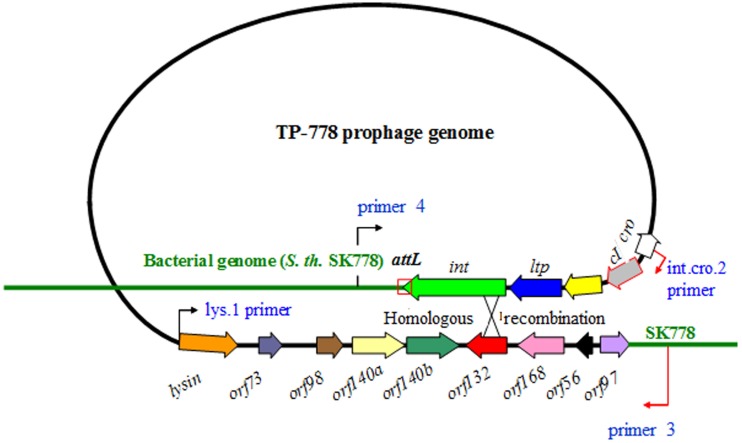
**Mechanism of excision of TP-778 prophage from its host's genome to yield phage TP-778L**. Prophage and host DNA are shown by black and green line, respectively. Genes are indicated by arrows. Binding sites of primers 4 and 3 (Bruttin et al., 1997 are shown). The region of predicted cross-over is indicated by a cross.

*S. thermophilus* SK778 could not be cured of its prophage. To find a host for lytic propagation, a set of 16 non-lysogenic *S. thermophilus* wild-type strains were tested for sensitivity to TP-778L. Only *S. thermophilus* strain B106, a host strain for propagation of temperate phage 7201 (Proux et al., 2002) which had been kindly provided by the University of Cork, Ireland, was found to allow plaque formation of TP-778L. Phage TP-778L was isolated as a plaque-purified, lytically propagated isolate. Its DNA sequence revealed that only a truncated integrase gene was present. Therefore, both host DNA regions flanking the prophage residing in the host genome were amplified by PCR and sequenced. Both flanking regions were found to be identical to *S. thermophilus* NDO3 DNA (Sun et al., 2011). The left region flanking the prophage's integrase gene contained a typical attachment site (Bruttin et al., 1997) overlapping with the 3′-end of the integrase gene of the prophage, which—in contrast to that of TP-778L—was complete. The right flanking region did not reveal an attachment site. Instead, a truncated integrase gene was seen, which showed high similarity to a phage remnant (Ventura et al., 2002). Comparison of the different integrase gene sequences indicated that excision of the prophage in case of TP-778L had occurred by recombination between the left complete and the right truncated integrase gene (Figure 6).

#### TP-EW

From an industrial yoghurt, we isolated lysogenic *S. thermophilus* strain EW carrying a prophage (called TP-EW). Upon induction with mitomycin C, a phage lysate of morphologically intact phage particles was obtained. Using a spot assay, TP-EW was shown to be able to productively infect *S. thermophilus* J34-6 (not shown). Restriction analysis with HindIII of DNA isolated from CsCl-purified phage particles revealed a pattern basically identical to TP-J34 DNA. Therefore, we consider this phage to be almost identical to TP-J34. However, two differences in the restriction pattern with respect to TP-J34 DNA were noticed (Figure 1A): the two fragments of TP-J34 of 5.0 and 4.4 kb were missing, instead, two new fragments of 1.7 and 6.0 kb were detected.

By Southern hybridization (Figure 1B) and DNA sequencing we could show that TP-EW DNA did not contain the 3 × 912 bp repeats found in the 4.4 kb fragment of TP-J34 DNA, but that it instead contained the fragment of 1.7 kb identical to the one of TP-J34L (Figure 4).

**Figure 7:**
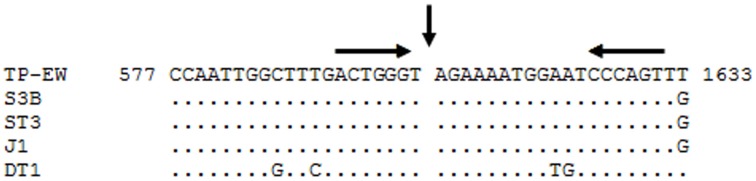
**Alignment of DNA sequences of *S. thermophilus* phages TP-EW, S3b (Acc. No. AF148561.1), ST3 (Acc. No. AF148565.1), J1 (Acc. No. AF148566.1), and DT1 (Acc. No. NC_002072) in the region surrounding the group-I-intron, present in all phage DNAs**. The splice site is indicated by the vertical arrow. Sequence differences are indicated. Two 6-bp inverted repeats are indicated by horizontal arrows above the DNA sequence. The numbers flanking the TP-EW sequence correspond to the nt positions within the *lys* gene of this phage.

The second differing restriction fragment of ca. 6 kb, when analyzed by additional restriction hydrolyses (not shown), appeared to be altered within the region of the lysin gene (*orf54*) with respect to TP-J34. A PCR with primers LYSup and LYSdown (Table 1) showed that TP-J34 DNA yielded a product of ca. 1.0 kb, while that of TP-EW DNA was ca. 1 kb larger (not shown). DNA sequencing and comparison with the TP-J34 DNA sequence indicated that the lysin gene of TP-EW contained an insertion of 1016 bp. BlastX analysis of the inserted sequence revealed an open reading frame encoding a protein of 205 amino acids with high homology to homing endonucleases (Lambowitz, 1993), indicating that the inserted sequence is a group I intron. Such introns have frequently been found in *S. thermophilus* phages to be located within the lysin gene (Foley et al., 2000). Comparison of the putative splice sites indicated high homology between *S. thermophilus* phages containing an intron in that position (Figure 7). Comparison of the DNA sequences flanking the insertion site of the intron with TP-J34 DNA sequence of that region revealed many deviations from TP-J34 sequence in the close vicinity, while the DNA sequences of TP-EW and TP-J34 were identical when they were more than a few hundred nucleotides apart from the insertion site.

Finally, for sequencing the *ltp*_TP−EW_ gene, we amplified a DNA region comprising the *ltp* gene plus the flanking regions by means of primers targeting sequences of TP-J34 genes *int* and *orf3*, respectively. The ca. 900 bp of nucleotide sequence obtained were 100% identical to those of TP-J34.

#### TP-DSM20617

*S. thermophilus* DSM20617 was obtained from the German type culture collection. It had been included in a screening for lysogenic *S. thermophilus* strains carrying *ltp*-expressing prophages (Sun, 2002). The DNA region of lysogenic strain *S. thermophilus* DSM20617 comprising *orf1* (integrase) through *orf6* (antirepressor) and defined by primers primer4 (left) and Mz12.R (right) was sequenced by primer walking. The sequence of ca. 3.7 kb was more than 99% identical to that of prophage TP-778 residing in *S. thermophilus* SK778. Only one base within orf1 (*int*), one base within orf2 (*ltp*), and two bases within orf 5 (*ant*) turned out to be different. Restriction analyses of DNA isolated from the phage lysate obtained by induction of the prophage did not reveal any similarities to restriction patterns of DNA isolated from TP-J34L and TP-778L, respectively (Figure S1A). Also, comparison of the HindIII and EcoRI patterns of TP-DSM20617 DNA with *in silico* generated patterns of 11 *S. thermophilus* phage genomes did not reveal any similarities (Figures S1B,C).

### Structural and functional aspects of *ltp* genes and products

**Figure 8:**
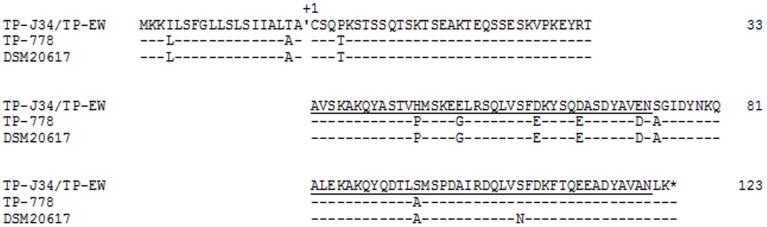
**Alignment of amino acid sequences of different Ltp proteins**. The cleavage site between signal sequence and mature protein is indicated. The first Cys of the mature TP-J34 lipoprotein is marked as +1. The two repeat regions are underlined. Amino acids identical to those of TP-J34 are indicated by “-.”

We compared the *ltp* gene products of the four phages (Figure 8). While Ltp_TP-J34_ and Ltp_TP-EW_ were identical, Ltp_TP-778_ and Ltp_TP-DSM20617_ differed in just one amino acid. However, both amino acid sequences of the mature proteins differed from that of mature Ltp_TP-J34_ in eight (Ltp_TP-778_) and nine (Ltp_TP-DSM20617_) positions, respectively. Most deviations were conservative substitutions (e.g., D vs. E) and were found within the first of the two repeat regions of the Ltp protein. We like to point out that in 2014 two protein sequences became available, which match the Ltp_TP-DSM20617_ sequence by 100%. One is from *S. thermophilus* prophage 20617 (Acc. no. CDG57923) and the other is from *S. thermophilus* M17PTZA496 (Acc. no. ETW90609).

To functionally compare Ltp_TP-778_ with Ltp_TP-J34_, we cloned *ltp*_TP-778_ in pMG36e, yielding plasmid pYAL1-3, exactly as *ltp*_TP-J34_ had been cloned to yield pXMS2 (Sun et al., 2006). After transformation of pYAL1-3 into *L. lactis* Bu2-60, the plating efficiencies of three lactococcal phages, which had already been tested against Ltp_TP-J34_ (Sun et al., 2006), were determined. Activity of Ltp_TP-778_ proved to be distinct from that of Ltp_TP-J34_: instead of strong inhibition of P008 as seen by Ltp_TP-J34_ almost no inhibition by Ltp_TP-778_ was recorded. Infection of phage P001, on the other hand was significantly impaired by Ltp_TP-778_, while Ltp_TP-J34_ did show almost no activity against P001 (Table 5).

**Table 5:** **Plating efficiencies (E.o.p.) of lactococcal phages on *L. lactis* Bu2-60 expressing plasmid-encoded copies of *ltp*_TP-J34_ or *ltp*_TP-778_**.

**Plasmid**	**Gene expressed**	**E.o.p**.		
		**P008**	**P335**	**P001**
pMG36e	–	1	1	1
pXMS2^a^	*ltp*_TP-J34_	10^−7^ to 10^−9^	0.7	0.7
pYAL1-3	*ltp*_TP-778_	0.6	0.35	0.0001–0.1^*^

*Means or ranges of at least three independently carried out assays are shown*.

* *Plaque sizes were significantly reduced*.

a *Data from Bebeacua et al. (2013)*.

To further broaden our knowledge on Ltp activity, we tested 11 additional virulent lactococcal phages by a semi-quantitative spot assay (Table 6). Based on their morphologies as determined by electron microscopy, these phages had been assigned to the three different species c2, 936, and P335, represented by the three phages described in Table 5. P008, P001, and P335 were included as controls in the assay. In general, the control phages were inhibited by the different Ltp proteins to extends similar as those presented in Table 5. However, the phages assigned to one species did not show homogeneous behavior. While two phages of the c2-species were not inhibited by Ltp_TP-J34_, three were strongly inhibited by this protein. On the other hand, one phage of this group was not inhibited by Ltp_TP-778_, while all other phages of this group were significantly inhibited. Such non-homogeneous behavior was also seen for the phages from the two other species. One should bear in mind that assignment to the species has to be considered preliminary. However, all phages assigned to the two species 936 and P335were inhibited to below detection level by the secreted, non-lipoprotein derivative UsLtp1, as has been described before for the three control phages (Bebeacua et al., 2013).

**Table 6:** **Semi-quantitative spottest for estimating the effects of different Ltp-proteins on infection of *L. lactis* Bu2-60 by different phage**.

**Phage**	**E.o.p. on *L. lactis* Bu2-60 expressing *ltp* gene**			
	**–**	***ltp*_TP-778_**	***ltp*_TP-J34_**	***usltp1*_TP-J34_**
**c2-SPECIES**				
P001	1^*^	10^−5^–10^−6^	1	10^−7^–10^−8^, turbid
P197	1	10^−6^–10^−7^	1	10^−6^–10^−7^, turbid
P220	1	10^−5^–10^−6^	1	10^−6^–10^−7^, turbid
P624	1 (10^9^–10^10^)	10^−5^–10^−6^, turbid	10^−7^–10^−8^	<10^−9^
P653	1 (10^9^–10^10^)	10^−4^–10^−5^, turbid	10^−6^–10^−7^, turbid	10^−6^– 10^−7^, turbid
P684	1 (10^9^–10^10^)	1	10^−5^–10^−6^	10^−5^–10^−6^, turbid
**936-SPECIES**				
P008	1	1	10^−7^–10^−8^, turbid	<10^−9^
P955	1	10^−6^–10^−7^	<10^−9^	<10^−9^
P957	1	1	10^−2^–10^−3^	<10^−9^
P983	1	1	0.1–1	<10^−9^
P993	1	10^−6^–10^−7^	<10^−9^	<10^−9^
P996	1	1	1	<10^−9^
**P335-SPECIES**				
P335	1	1	1	<10^−9^
P615	1	1	<10^−9^	<10^−9^

* *If not indicated, titers of lysates were >10^10^ pfu per ml. Deviating titers are shown in brackets*.

## Discussion

Our screening for Ltp-expressing prophages in *S. thermophilus* yielded just four different phages, three of which (TP-J34, TP-EW, TP-778) can be assigned to the Sfi11 sub-species species of *S. thermophilus* phages (Proux et al., 2002; Quiberoni et al., 2010), since they are *pac*-type phages and their genome sequences show high similarities to phages Sfi11 and O1205. The fourth phage, TP-DSM20617 cannot be classified due to lack of information on its genome. The three phages, TP-J34 and TP-EW on one hand and TP-778 on the other, appear to represent two different lines within the Sfi11 sub-species, with the major difference between the two types being lack of homology between the genes within the “replication” module. Other minor differences are seen within the modules of “DNA-packaging,” “tail morphogenesis,” and “lysogenic conversion.” The exchange of entire functional modules appears to be the general mechanism of recombination between bacteriophages (Lucchini et al., 1998). Such exchange is easily accomplished without impairing functionality of the phage, especially when interaction with proteins of other modules does not occur. This is the case with the proteins of the “replication” as well as the “lysogenic conversion” module. The “DNA packaging” module consists of two proteins only, the small (TerS) and the large terminase (TerL) units. The portal protein, encoded by the gene immediately following that of the large terminase, may be considered part of this module, however it also plays a critical role in head assembly (Padilla-Sanchez et al., 2013). The lack of similarity within the “DNA packaging” module only affects the N-terminal and central regions of TerS, which are involved in DNA binding and oligomerization, respectively (Sun et al., 2012). The C-terminal part, which is involved in interaction with the portal protein, is absolutely identical between TP-J34 and TP-778L. Thus, functionality defined as productive interaction with other components of the module is apparently not impaired by the alterations affecting TerS. The fact that both phages are *pac*-type phages and show high genome similarities to phages Sfi11 and O1205 confirms this finding. The last region of divergence between TP-J34 and TP-778L DNA concerns the “tail morphogenesis” module. Compared to the TP-J34 module, *orf*s *45* and *48* appear to be fused to form the one large *orf42* of TP-778L. The gene product of *orf45* is characterized by a Lyz2 (Nambu et al., 1999) and a CHAP-domain (Bateman and Rawlings, 2003), indicating involvement in peptidoglycan hydrolysis during infection following adsorption. The gene product of *orf48* appears to be the receptor binding protein, containing a domain which is found in galactose-binding proteins (Gaskell et al., 1995). These three domains are found in the *orf42* gene product of TP-778L. It appears that both functions, which are required at the first steps of infection in TP-778, are combined in just one protein. This is not too surprising, since proteins encoded by genes with adjacent positions on the genetic map may also be in close contact within the structures formed. A fact that has been the basis for successful “block cloning” applied for elucidation of tail sub-structures (Campanacci et al., 2010).

The *orf48* gene product, containing the three 912 bp repeats, appears to be either physically unstable or inactive in the tail assembly process. The few intact phage particles found after induction may arise from recombinational loss of the repeats occurring during replication: the few functional copies of Orf48 produced may initiate successful tail assembly. If TP-J34 DNA lacking the 912 bp repeat is packaged into such phage particles, TP-J34L phage particles are produced. The observed very low efficiency of plating for phage lysates resulting from induction of the prophage (Neve et al., 2003), even if they contained just one repeat may be due to phenotypic mixing (Streisinger, 1956), i.e., packaging of DNA into phage particles which are not derived from that DNA.

The 912 bp repeat shows DNA sequence homology to its flanking regions. However, an internal region of ca. 450 bp of the 912 bp repeat does not show homology to the flanking DNA or to other regions of TP-J34 DNA, which may indicate that this DNA region had been introduced by horizontal gene transfer. BlastN analysis revealed 80% sequence identity over the 450 bp to the host specificity gene of *S. thermophilus* bacteriophage DT2 (Duplessis and Moineau, 2001), and BlastX revealed 75% sequence similarity (*E*-value 2e-60) over 150 amino acids of the product of that gene. One may speculate that the DNA region has been obtained by horizontal gene transfer from a not yet identified phage with homology to phage DT2 in this genome region.

Horizontal gene transfer is apparently also responsible for the distribution of *ltp* genes, encoding a sie lipoprotein, among strains and bacteriophages of Gram-positive bacteria (Sun et al., 2006). The members of this family of “host cell surface-exposed lipoproteins” (Marchler-Bauer et al., 2011) are found scattered within annotated genomes of bacteriophage and bacteria (Sun et al., 2006). This would argue for *ltp* to be a member of the so called “morons,” genes inserted into prophage genomes by horizontal gene transfer which provide some benefit to the host (Cumby et al., 2012). Further additional evidence for the “moron” character of *ltp* like presence of promoter and terminator will be presented elsewhere (Koberg et al., in preparation). The fact that the few temperate *S. thermophilus* phage harboring *ltp* are all very closely related indicates that horizontal transfer of an *ltp* gene into *S. thermophilus* phage occurred just once. The genome deviations seen among the three phages TP-J34, TP-778, and TP-EW should therefore have occurred after *ltp* had been acquired.

The differences in amino acid sequences and activities seen between plasmid-expressed Ltp_TP-J34_ and Ltp_TP-778_ confirm our recent data on Ltp_TP-J34_ structure (Bebeacua et al., 2013), which indicated that the repeat domains are those responsible for super infection exclusion by interaction with the TMP of the super infecting phage and that the negatively charged amino acids in this region are important for interacting with the positively charged C-terminal end region of the P008 TMP. The deviations from Ltp_TP-J34_ seen in the amino acid sequences of the Ltp_TP-778_ repeat domain are mostly conservative. It is intriguing that with one exception the charges are not changed by the deviations. At this point it would just be speculation that the one change from negatively charged Glu to neutral Gly (see Figure 8) would be responsible for the functional differences. Another candidate for this difference could be the amino acid change from His to Pro (see Figure 8). However, this exchange does not affect a helix but just a ß-turn within the first repeat domain.

When discussing the potential effects on interaction with TMP of the amino acid exchanges seen between Ltp_TP-J34_ and Ltp_TP-778_, one should bear in mind that no genome sequence is available for lactococcal phage P001, a member of the c2-species. In the available genome sequence of lactococcal phage c2, however, no TMP is annotated (Lubbers et al., 1995). This is apparently due to the fact that phage c2 uses the host “phage infection protein” Pip for adsorption and DNA-injection (Monteville et al., 1994). In phage c2, gene 110 encoding the “tail adsorption protein” should be the TMP of phage c2. This protein would not need to encompass the pore-forming function, since Pip provides this function. The fact that the secreted soluble UsLtp_TP-J34_ is considerably less active against most phages attributed to the c2-species apparently underlines the peculiar situation of c2-phages with respect to TMP. With UsLtp_TP-J34_ at hand, we may be able to test whether the “tail adsorption protein” is in fact the TMP of c2. At this stage, we can just notice that the C-terminal end of the c2 “tail adsorption protein” is positively charged, which is in agreement with the proposed binding site of Ltp_TP-J34_ in TMP of P008 (Bebeacua et al., 2013).

To conclude, in this communication we could show that amino acid deviations seen between Ltp_TP-J34_ and Ltp_TP-778_ are apparently responsible for differences seen in the biological activities of both proteins. These deviations provide some clues on how to further study interaction between Ltp and TMP in more detail. Our data also show that phages TP-J34, TP-778, and TP-EW belong to the Sfi11 sub-species of *S. thermophilus* phages. The close relatedness of the three phages argues for acquisition of *ltp* prior to formation of the three phages from a common ancestor.

### Conflict of interest statement

The authors declare that the research was conducted in the absence of any commercial or financial relationships that could be construed as a potential conflict of interest.
