# Supplementary material for: Temperate Streptococcus thermophilus phages expressing superinfection exclusion proteins of the Ltp type
Source: Front Microbiol. 2014 Mar 13;5:98. doi: 10.3389/fmicb.2014.00098 (PMC3952083; doi:10.3389/fmicb.2014.00098)
Supplement: Supplementary file 1 [file Presentation1.PPTX]

## Slide 1
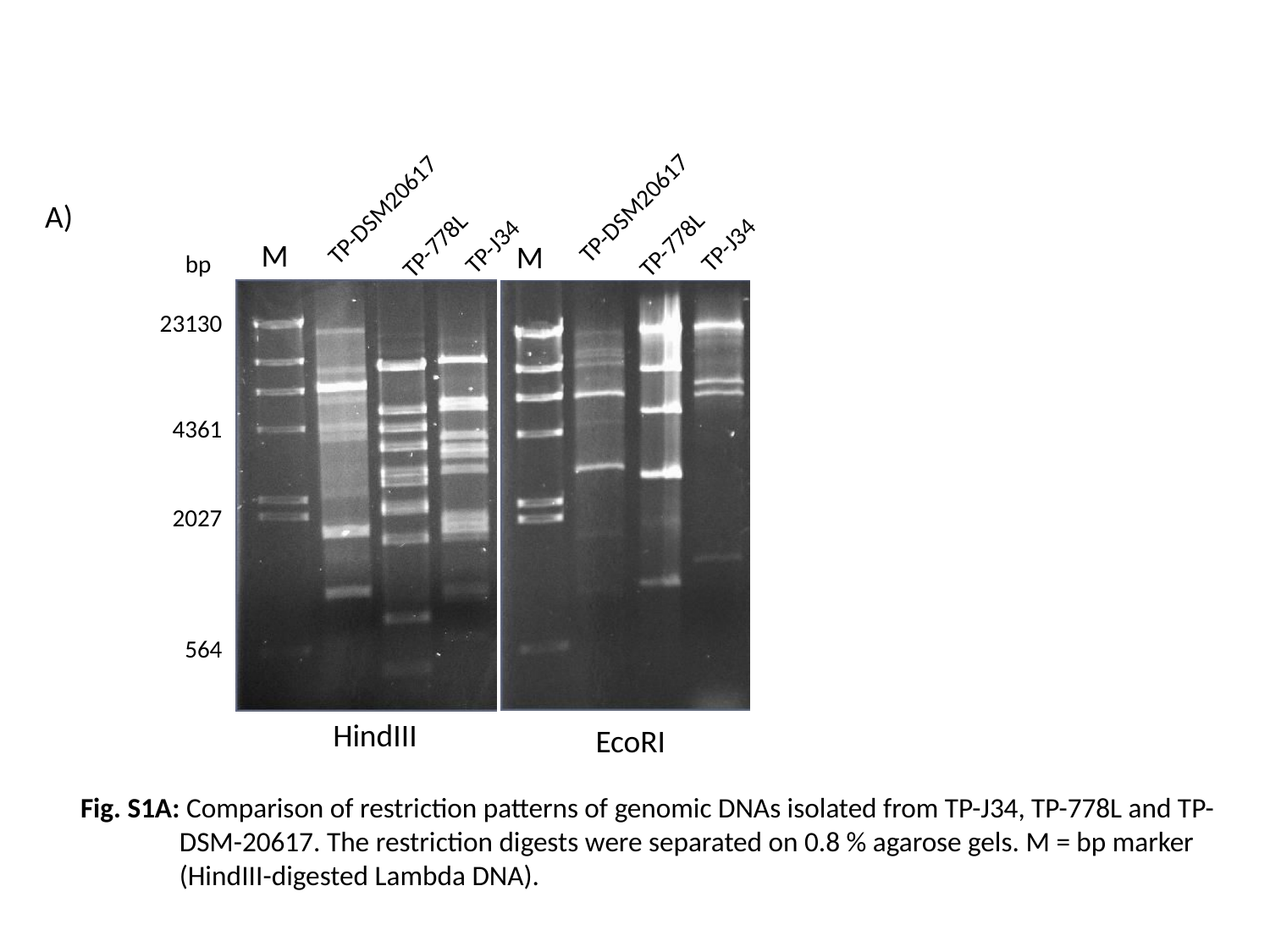

TP-DSM20617
TP-DSM20617
TP-778L
TP-778L
TP-J34
TP-J34
M
M
HindIII
EcoRI
bp
23130
4361
2027
564
A)
Fig. S1A: Comparison of restriction patterns of genomic DNAs isolated from TP-J34, TP-778L and TP-DSM-20617. The restriction digests were separated on 0.8 % agarose gels. M = bp marker (HindIII-digested Lambda DNA).

## Slide 2
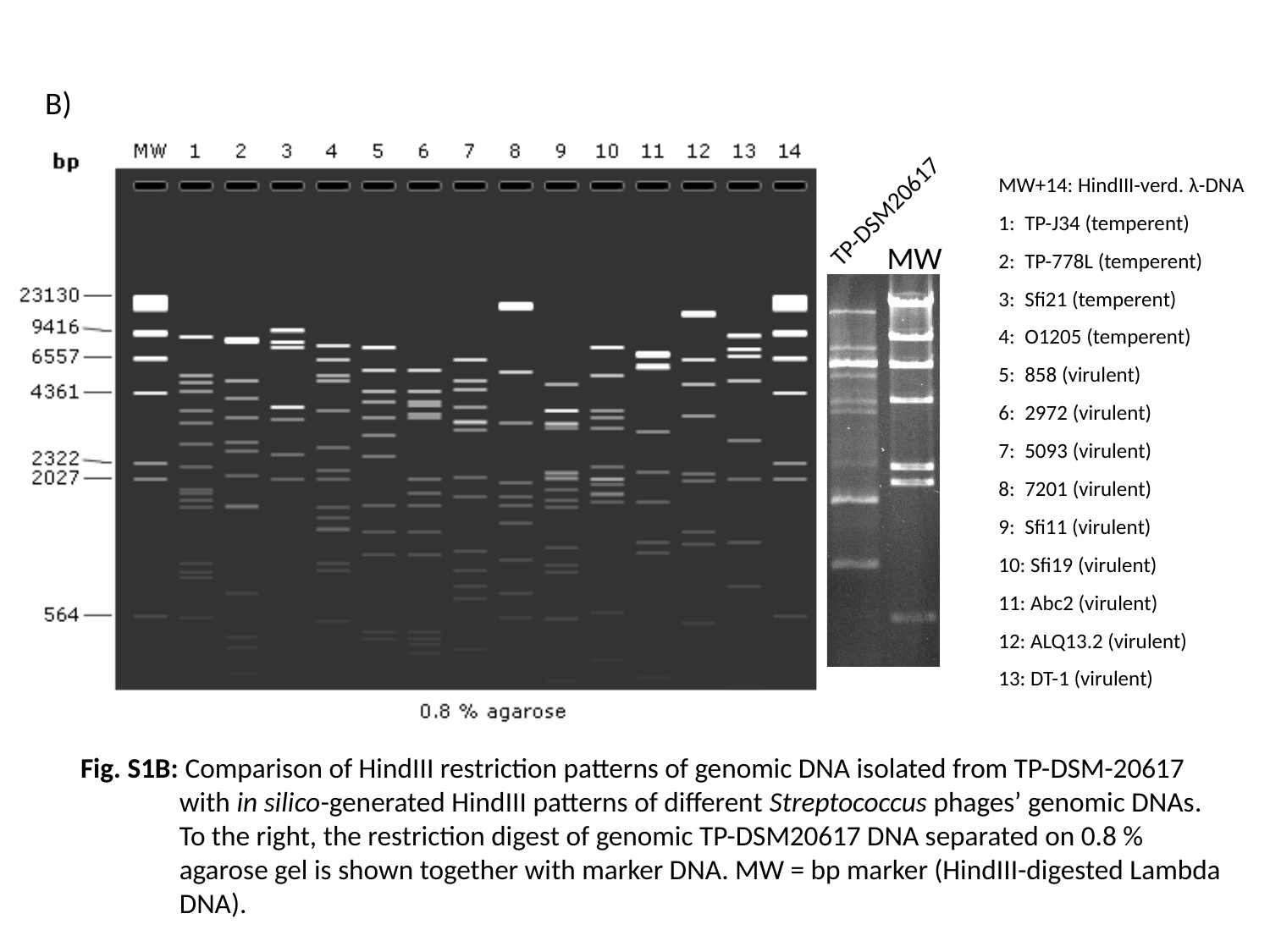

B)
MW+14: HindIII-verd. λ-DNA
1: TP-J34 (temperent)
2: TP-778L (temperent)
3: Sfi21 (temperent)
4: O1205 (temperent)
5: 858 (virulent)
6: 2972 (virulent)
7: 5093 (virulent)
8: 7201 (virulent)
9: Sfi11 (virulent)
10: Sfi19 (virulent)
11: Abc2 (virulent)
12: ALQ13.2 (virulent)
13: DT-1 (virulent)
TP-DSM20617
MW
Fig. S1B: Comparison of HindIII restriction patterns of genomic DNA isolated from TP-DSM-20617 with in silico-generated HindIII patterns of different Streptococcus phages’ genomic DNAs. To the right, the restriction digest of genomic TP-DSM20617 DNA separated on 0.8 % agarose gel is shown together with marker DNA. MW = bp marker (HindIII-digested Lambda DNA).

## Slide 3
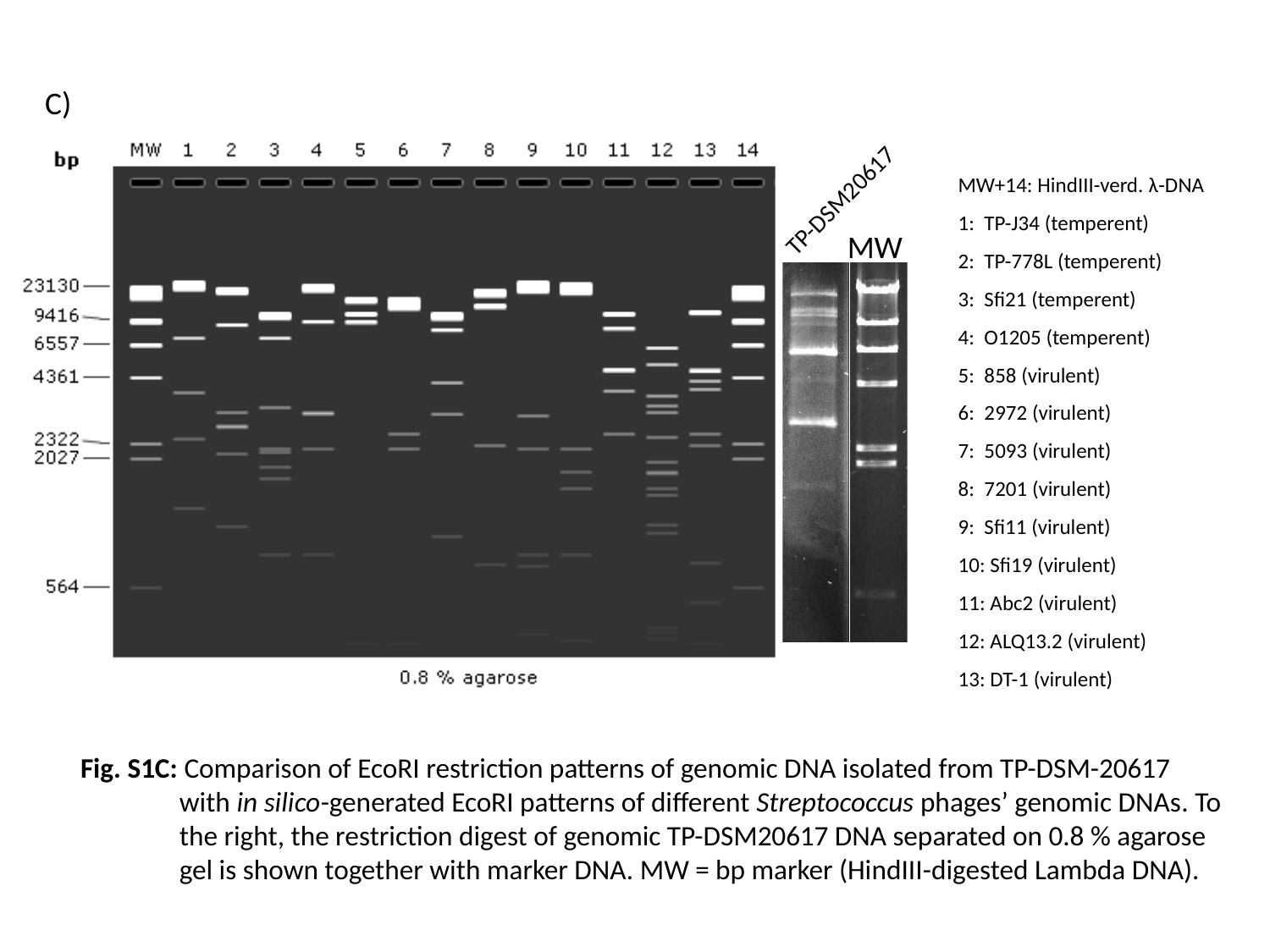

C)
MW+14: HindIII-verd. λ-DNA
1: TP-J34 (temperent)
2: TP-778L (temperent)
3: Sfi21 (temperent)
4: O1205 (temperent)
5: 858 (virulent)
6: 2972 (virulent)
7: 5093 (virulent)
8: 7201 (virulent)
9: Sfi11 (virulent)
10: Sfi19 (virulent)
11: Abc2 (virulent)
12: ALQ13.2 (virulent)
13: DT-1 (virulent)
TP-DSM20617
MW
Fig. S1C: Comparison of EcoRI restriction patterns of genomic DNA isolated from TP-DSM-20617 with in silico-generated EcoRI patterns of different Streptococcus phages’ genomic DNAs. To the right, the restriction digest of genomic TP-DSM20617 DNA separated on 0.8 % agarose gel is shown together with marker DNA. MW = bp marker (HindIII-digested Lambda DNA).
